# [2-Acet­oxy-3-(naphthalen-1-yl­oxy)prop­yl](propan-2-yl)aza­nium chloride monohydrate

**DOI:** 10.1107/S1600536813005515

**Published:** 2013-03-09

**Authors:** Yuan-Yuan Liu, Guang-Hui Xu, Zheng-Jie Li, Hong-Yu Xu, Chang-Qing Gu

**Affiliations:** a Department of Chemical and Pharmaceutical Engineering, Southeast University ChengXian College, Nanjing 210088, People’s Republic of China

## Abstract

The title compound, C_18_H_24_NO_3_^+^·Cl^−^·H_2_O, was synthesized by the reaction of propranolol hydro­chloride with acetyl chloride in chloro­form followed by slow evaporation in air. In the cation, the dihedral angle between the planes of the naphthalene ring system and the acetate group is 71.1 (2)°. An intra­molecular N—H⋯O hydrogen bond results in the formation of a non-planar pseudo-ring, with the ether O and the H atom displaced by −1.328 (2) and 0.65 Å, respectively, from the plane of the other ring atoms. The cation and anion are linked by an N—H⋯Cl hydrogen bond. The water molecule is linked to a methyl H atom by C—H⋯O hydrogen bond.

## Related literature

The applications of the title compound, see: Barbosa *et al.* (2010 ▶). For the synthetic procedure, see: Irwin & Belaid (1987 ▶). For bond-length data, see: Allen *et al.* (1987 ▶).
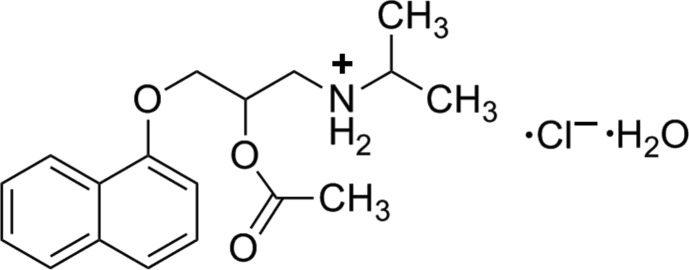


## Experimental

### 

#### Crystal data

C_18_H_24_NO_3_^+^·Cl^−^·H_2_O
*M* *_r_* = 355.85
Monoclinic, 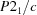

*a* = 15.559 (3) Å
*b* = 8.2120 (16) Å
*c* = 14.665 (3) Å
β = 93.23 (3)°
*V* = 1870.8 (6) Å^3^
*Z* = 4
Mo *K*α radiation
μ = 0.23 mm^−1^
*T* = 293 K
0.30 × 0.20 × 0.10 mm

#### Data collection

Enraf–Nonius CAD-4 diffractometer
Absorption correction: ψ scan (North *et al.*, 1968 ▶) *T*_min_ = 0.936, *T*_max_ = 0.978
3424 measured reflections
3424 independent reflections
1948 reflections with *I* > 2σ(*I*)
3 standard reflections every 200 reflections intensity decay: 1%

#### Refinement

*R*[*F* ^2^ > 2σ(*F* ^2^)] = 0.070
*wR*(*F* ^2^) = 0.200
*S* = 0.99
3424 reflections
219 parameters
1 restraint
H-atom parameters constrained
Δρ_max_ = 0.60 e Å^−3^
Δρ_min_ = −0.28 e Å^−3^

### 

Data collection: *CAD-4 Software* (Enraf–Nonius, 1985 ▶); cell refinement: *CAD-4 Software*; data reduction: *XCAD4* (Harms & Wocadlo, 1995 ▶); program(s) used to solve structure: *SHELXS97* (Sheldrick, 2008 ▶); program(s) used to refine structure: *SHELXL97* (Sheldrick, 2008 ▶); molecular graphics: *SHELXTL* (Sheldrick, 2008 ▶); software used to prepare material for publication: *SHELXTL*.

## Supplementary Material

Crystal structure: contains datablock(s) I, global. DOI: 10.1107/S1600536813005515/bq2383sup1.cif

Structure factors: contains datablock(s) I. DOI: 10.1107/S1600536813005515/bq2383Isup2.hkl

Supplementary material file. DOI: 10.1107/S1600536813005515/bq2383Isup3.cml

Additional supplementary materials:  crystallographic information; 3D view; checkCIF report

## Figures and Tables

**Table 1:** Hydrogen-bond geometry (Å, °)

*D*—H⋯*A*	*D*—H	H⋯*A*	*D*⋯*A*	*D*—H⋯*A*
N—H0*A*⋯Cl	0.90	2.37	3.194 (3)	152
N—H0*A*⋯O2	0.90	2.59	2.948 (3)	105
C15—H15*B*⋯O1*W* ^i^	0.96	2.53	3.469 (9)	166

Symmetry code: (i) 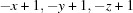.

**Fig. 1.**
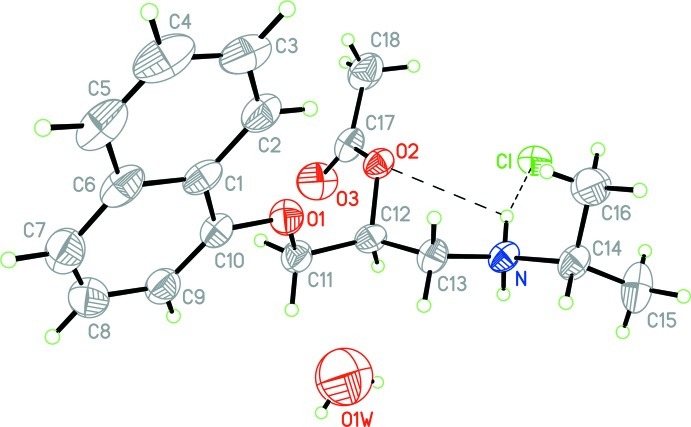
The molecular structure of (I), with the atom-numbering scheme. Displacement ellipsoids are drawn at the 50% probability level. Hydrogen bonds are shown by dashed lines.

**Fig. 2.**
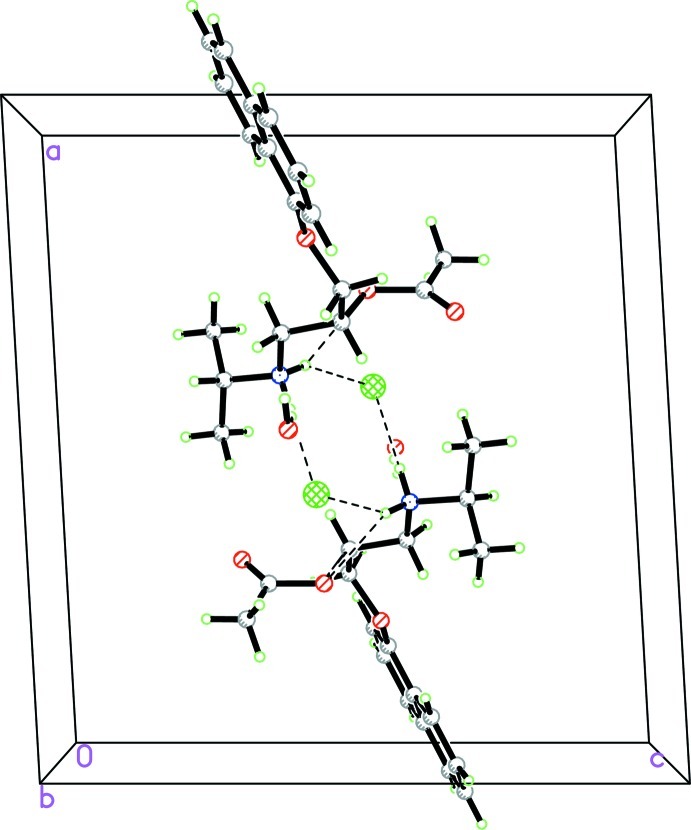
A packing diagram of (I). Hydrogen bonds are shown by dashed lines.

## supplementary crystallographic information

### Comment

Propranolol hydrochloride is an important beta-adrenergic blocking agent, and used in the treatment of hypertension and cardiovascular disorders. Molecular modifications of propranolol are varied with examples in the amino-substituent, side-chain variants and nuclear changes including all mono ring-hydroxylated products (Irwin *et al.*, 1987). Here, the title compound, (I), an important propranolol derivative, was synthesized by the reaction of propranolol hydrochloride with acetyl chloride in chloroform, and we report the crystal structure of (I).

In the molecule of (I), (Fig.1), the bond lengths (Allen *et al.*, 1987) and angles are within normal ranges. The dihedral angle of naphthalene ring system and acetate group is 71.11 °. The amino hydrogen is linked to chloride and oxygen atoms by the intramolecular N—H···Cl and N—H···O hydrogen bonds, respectively, which seem to be very effective in the stabilization of the crystal structure. The intramolecular N—H···O hydrogen bond results in the formation of one non-planar pseudo ring (O2/C12/C13/N/H0A), with O2 and H0A atoms displaced by -1.328 Å and 0.65 Å, respectively, from the plane of the other ring atoms.

### Experimental

The title compound, (I) was prepared by the literature method (Irwin *et al.*, 1987). Crystals suitable for X-ray analysis were obtained by dissolving (I) (0.5 g) in methanol (20 ml) and evaporating the solvent slowly at room temperature for about 10 d.

### Refinement

H atoms were positioned geometrically, with N—H = 0.90 Å (for NH), C—H = 0.93, 0.96, 0.97 and 0.98 Å for aromatic, methyl, methylene and methine H, respectively, and constrained to ride on their parent atoms, with *U*_iso_(H) = *xU*_eq_(C), where *x* = 1.2 for aromatic H, and *x* = 1.5 for other H.

### Crystal data

**Table:**

C_18_H_24_NO_3_^+^·Cl^−^·H_2_O	*F*(000) = 760
*M**_r_* = 355.85	*D*_x_ = 1.263 Mg m^−^^3^
Monoclinic, *P*2_1_/*c*	Mo *K*α radiation, λ = 0.71073 Å
Hall symbol: -P 2ybc	Cell parameters from 25 reflections
*a* = 15.559 (3) Å	θ = 9–13°
*b* = 8.2120 (16) Å	µ = 0.23 mm^−^^1^
*c* = 14.665 (3) Å	*T* = 293 K
β = 93.23 (3)°	Block, yellow
*V* = 1870.8 (6) Å^3^	0.30 × 0.20 × 0.10 mm
*Z* = 4	

### Data collection

**Table:**

Enraf–Nonius CAD-4 diffractometer	1948 reflections with *I* > 2σ(*I*)
Radiation source: fine-focus sealed tube	*R*_int_ = 0.000
Graphite monochromator	θ_max_ = 25.4°, θ_min_ = 1.3°
ω/2θ scans	*h* = −18→18
Absorption correction: ψ scan (North *et al.*, 1968)	*k* = 0→9
*T*_min_ = 0.936, *T*_max_ = 0.978	*l* = 0→17
3424 measured reflections	3 standard reflections every 200 reflections
3424 independent reflections	intensity decay: 1%

### Refinement

**Table:**

Refinement on *F*^2^	Primary atom site location: structure-invariant direct methods
Least-squares matrix: full	Secondary atom site location: difference Fourier map
*R*[*F*^2^ > 2σ(*F*^2^)] = 0.070	Hydrogen site location: inferred from neighbouring sites
*wR*(*F*^2^) = 0.200	H-atom parameters constrained
*S* = 0.99	*w* = 1/[σ^2^(*F*_o_^2^) + (0.1097*P*)^2^] where *P* = (*F*_o_^2^ + 2*F*_c_^2^)/3
3424 reflections	(Δ/σ)_max_ = 0.001
219 parameters	Δρ_max_ = 0.60 e Å^−^^3^
1 restraint	Δρ_min_ = −0.28 e Å^−^^3^

### Special details

**Table:**

Geometry. All e.s.d.'s (except the e.s.d. in the dihedral angle between two l.s. planes) are estimated using the full covariance matrix. The cell e.s.d.'s are taken into account individually in the estimation of e.s.d.'s in distances, angles and torsion angles; correlations between e.s.d.'s in cell parameters are only used when they are defined by crystal symmetry. An approximate (isotropic) treatment of cell e.s.d.'s is used for estimating e.s.d.'s involving l.s. planes.
Refinement. Refinement of *F*^2^ against ALL reflections. The weighted *R*-factor *wR* and goodness of fit *S* are based on *F*^2^, conventional *R*-factors *R* are based on *F*, with *F* set to zero for negative *F*^2^. The threshold expression of *F*^2^ > σ(*F*^2^) is used only for calculating *R*-factors(gt) *etc*. and is not relevant to the choice of reflections for refinement. *R*-factors based on *F*^2^ are statistically about twice as large as those based on *F*, and *R*- factors based on ALL data will be even larger.

### Fractional atomic coordinates and isotropic or equivalent isotropic displacement parameters (Å^2^)

**Table:**

	*x*	*y*	*z*	*U*_iso_*/*U*_eq_	
N	0.40172 (17)	0.4306 (4)	0.60092 (17)	0.0541 (7)	
H0A	0.3855	0.5068	0.5593	0.065*	
H0B	0.4541	0.3943	0.5874	0.065*	
O1	0.20414 (15)	0.0867 (3)	0.54378 (16)	0.0606 (7)	
C1	0.0783 (2)	−0.0245 (5)	0.5948 (2)	0.0591 (10)	
O2	0.27260 (14)	0.3625 (3)	0.45087 (15)	0.0553 (6)	
C2	0.0499 (2)	0.1326 (6)	0.6206 (2)	0.0669 (11)	
H2A	0.0842	0.2231	0.6110	0.080*	
O3	0.3081 (2)	0.2709 (4)	0.31387 (19)	0.0930 (10)	
C3	−0.0272 (3)	0.1522 (7)	0.6592 (3)	0.0810 (13)	
H3A	−0.0450	0.2555	0.6758	0.097*	
C4	−0.0788 (3)	0.0189 (9)	0.6735 (3)	0.0974 (17)	
H4A	−0.1314	0.0343	0.6993	0.117*	
C5	−0.0547 (3)	−0.1334 (8)	0.6510 (3)	0.0886 (15)	
H5A	−0.0908	−0.2206	0.6619	0.106*	
C6	0.0248 (3)	−0.1623 (6)	0.6110 (3)	0.0714 (12)	
C7	0.0532 (3)	−0.3184 (6)	0.5867 (3)	0.0811 (13)	
H7A	0.0194	−0.4091	0.5972	0.097*	
C8	0.1295 (3)	−0.3368 (6)	0.5482 (3)	0.0821 (13)	
H8A	0.1473	−0.4406	0.5322	0.099*	
C9	0.1817 (3)	−0.2049 (5)	0.5320 (3)	0.0652 (10)	
H9A	0.2338	−0.2211	0.5052	0.078*	
C10	0.1576 (2)	−0.0522 (5)	0.5546 (2)	0.0538 (9)	
C11	0.2810 (2)	0.0738 (5)	0.4924 (3)	0.0592 (9)	
H11A	0.2657	0.0439	0.4296	0.071*	
H11B	0.3192	−0.0088	0.5191	0.071*	
C12	0.3240 (2)	0.2361 (4)	0.4961 (2)	0.0537 (9)	
H12A	0.3791	0.2280	0.4672	0.064*	
C13	0.3402 (2)	0.2938 (5)	0.5934 (2)	0.0582 (9)	
H13A	0.2861	0.3276	0.6172	0.070*	
H13B	0.3626	0.2039	0.6305	0.070*	
C14	0.4085 (2)	0.5085 (5)	0.6944 (2)	0.0603 (10)	
H14A	0.4113	0.4211	0.7400	0.072*	
C15	0.4901 (3)	0.6048 (6)	0.7055 (3)	0.1012 (17)	
H15A	0.4967	0.6463	0.7666	0.152*	
H15B	0.4879	0.6939	0.6630	0.152*	
H15C	0.5381	0.5359	0.6938	0.152*	
C16	0.3301 (3)	0.6086 (6)	0.7096 (3)	0.0930 (15)	
H16A	0.3348	0.6548	0.7698	0.140*	
H16B	0.2798	0.5409	0.7035	0.140*	
H16C	0.3255	0.6945	0.6652	0.140*	
C17	0.2726 (2)	0.3674 (5)	0.3586 (2)	0.0626 (10)	
C18	0.2213 (3)	0.5119 (6)	0.3213 (3)	0.0890 (14)	
H18A	0.2218	0.5128	0.2559	0.134*	
H18B	0.2465	0.6108	0.3453	0.134*	
H18C	0.1630	0.5037	0.3391	0.134*	
Cl	0.41641 (6)	0.70545 (13)	0.44852 (7)	0.0688 (4)	
O1W	0.5140 (4)	0.0297 (9)	0.4132 (5)	0.252 (3)	
H1WB	0.5328	−0.0674	0.4172	0.302*	
H1WA	0.5574	0.0922	0.4113	0.302*	

### Atomic displacement parameters (Å^2^)

**Table:**

	*U*^11^	*U*^22^	*U*^33^	*U*^12^	*U*^13^	*U*^23^
N	0.0495 (16)	0.0682 (19)	0.0441 (15)	−0.0011 (15)	−0.0008 (12)	−0.0002 (14)
O1	0.0526 (14)	0.0639 (16)	0.0663 (16)	−0.0022 (13)	0.0111 (12)	−0.0077 (13)
C1	0.050 (2)	0.085 (3)	0.0401 (18)	0.002 (2)	−0.0130 (16)	0.0034 (18)
O2	0.0534 (14)	0.0682 (16)	0.0438 (13)	0.0015 (12)	−0.0022 (10)	−0.0029 (11)
C2	0.054 (2)	0.090 (3)	0.056 (2)	0.002 (2)	−0.0078 (17)	−0.008 (2)
O3	0.110 (3)	0.121 (3)	0.0496 (16)	0.022 (2)	0.0121 (16)	−0.0121 (17)
C3	0.056 (3)	0.122 (4)	0.064 (3)	0.021 (3)	−0.004 (2)	−0.008 (3)
C4	0.058 (3)	0.165 (6)	0.069 (3)	0.010 (4)	0.007 (2)	0.010 (3)
C5	0.056 (3)	0.138 (5)	0.071 (3)	−0.019 (3)	−0.004 (2)	0.019 (3)
C6	0.053 (2)	0.106 (4)	0.054 (2)	−0.012 (2)	−0.0072 (17)	0.018 (2)
C7	0.086 (3)	0.082 (3)	0.074 (3)	−0.021 (3)	−0.010 (2)	0.018 (2)
C8	0.089 (3)	0.074 (3)	0.082 (3)	−0.001 (3)	−0.008 (3)	0.008 (2)
C9	0.061 (2)	0.072 (3)	0.061 (2)	−0.003 (2)	−0.0072 (18)	0.001 (2)
C10	0.0479 (19)	0.066 (3)	0.0473 (19)	−0.0038 (19)	−0.0015 (15)	−0.0021 (17)
C11	0.051 (2)	0.063 (2)	0.063 (2)	−0.0006 (18)	0.0019 (17)	−0.0068 (18)
C12	0.0424 (18)	0.066 (2)	0.053 (2)	0.0026 (17)	0.0037 (15)	0.0012 (17)
C13	0.059 (2)	0.067 (2)	0.049 (2)	−0.008 (2)	−0.0005 (16)	−0.0014 (18)
C14	0.064 (2)	0.076 (3)	0.0399 (18)	−0.002 (2)	−0.0031 (16)	−0.0029 (18)
C15	0.100 (4)	0.113 (4)	0.090 (3)	−0.035 (3)	0.004 (3)	−0.036 (3)
C16	0.090 (3)	0.108 (4)	0.079 (3)	0.028 (3)	−0.009 (2)	−0.027 (3)
C17	0.058 (2)	0.081 (3)	0.049 (2)	−0.016 (2)	−0.0001 (17)	0.001 (2)
C18	0.093 (3)	0.099 (3)	0.073 (3)	−0.012 (3)	−0.021 (2)	0.016 (3)
Cl	0.0648 (6)	0.0687 (7)	0.0732 (7)	0.0056 (5)	0.0059 (5)	0.0091 (5)
O1W	0.240 (7)	0.227 (7)	0.286 (8)	−0.022 (6)	−0.014 (7)	0.026 (6)

### Geometric parameters (Å, º)

**Table:**

N—C13	1.476 (4)	C9—C10	1.355 (5)
N—C14	1.511 (4)	C9—H9A	0.9300
N—H0A	0.9000	C11—C12	1.491 (5)
N—H0B	0.9000	C11—H11A	0.9700
O1—C10	1.365 (4)	C11—H11B	0.9700
O1—C11	1.452 (4)	C12—C13	1.511 (5)
C1—C10	1.416 (5)	C12—H12A	0.9800
C1—C2	1.422 (5)	C13—H13A	0.9700
C1—C6	1.432 (5)	C13—H13B	0.9700
O2—C17	1.353 (4)	C14—C15	1.498 (5)
O2—C12	1.448 (4)	C14—C16	1.498 (5)
C2—C3	1.364 (5)	C14—H14A	0.9800
C2—H2A	0.9300	C15—H15A	0.9600
O3—C17	1.186 (5)	C15—H15B	0.9600
C3—C4	1.380 (7)	C15—H15C	0.9600
C3—H3A	0.9300	C16—H16A	0.9600
C4—C5	1.352 (7)	C16—H16B	0.9600
C4—H4A	0.9300	C16—H16C	0.9600
C5—C6	1.419 (6)	C17—C18	1.515 (6)
C5—H5A	0.9300	C18—H18A	0.9600
C6—C7	1.409 (6)	C18—H18B	0.9600
C7—C8	1.351 (6)	C18—H18C	0.9600
C7—H7A	0.9300	O1W—H1WB	0.8500
C8—C9	1.382 (6)	O1W—H1WA	0.8500
C8—H8A	0.9300		
			
C13—N—C14	113.7 (3)	C12—C11—H11B	110.3
C13—N—H0A	108.8	H11A—C11—H11B	108.5
C14—N—H0A	108.8	O2—C12—C11	112.9 (3)
C13—N—H0B	108.8	O2—C12—C13	105.5 (3)
C14—N—H0B	108.8	C11—C12—C13	111.4 (3)
H0A—N—H0B	107.7	O2—C12—H12A	109.0
C10—O1—C11	117.2 (3)	C11—C12—H12A	109.0
C10—C1—C2	123.2 (4)	C13—C12—H12A	109.0
C10—C1—C6	118.1 (4)	N—C13—C12	112.5 (3)
C2—C1—C6	118.7 (4)	N—C13—H13A	109.1
C17—O2—C12	116.6 (3)	C12—C13—H13A	109.1
C3—C2—C1	120.7 (4)	N—C13—H13B	109.1
C3—C2—H2A	119.6	C12—C13—H13B	109.1
C1—C2—H2A	119.6	H13A—C13—H13B	107.8
C2—C3—C4	120.2 (5)	C15—C14—C16	112.7 (4)
C2—C3—H3A	119.9	C15—C14—N	109.7 (3)
C4—C3—H3A	119.9	C16—C14—N	110.6 (3)
C5—C4—C3	121.6 (5)	C15—C14—H14A	107.9
C5—C4—H4A	119.2	C16—C14—H14A	107.9
C3—C4—H4A	119.2	N—C14—H14A	107.9
C4—C5—C6	121.0 (5)	C14—C15—H15A	109.5
C4—C5—H5A	119.5	C14—C15—H15B	109.5
C6—C5—H5A	119.5	H15A—C15—H15B	109.5
C7—C6—C5	123.4 (5)	C14—C15—H15C	109.5
C7—C6—C1	118.9 (4)	H15A—C15—H15C	109.5
C5—C6—C1	117.7 (5)	H15B—C15—H15C	109.5
C8—C7—C6	120.2 (4)	C14—C16—H16A	109.5
C8—C7—H7A	119.9	C14—C16—H16B	109.5
C6—C7—H7A	119.9	H16A—C16—H16B	109.5
C7—C8—C9	121.5 (5)	C14—C16—H16C	109.5
C7—C8—H8A	119.3	H16A—C16—H16C	109.5
C9—C8—H8A	119.3	H16B—C16—H16C	109.5
C10—C9—C8	120.7 (4)	O3—C17—O2	124.0 (4)
C10—C9—H9A	119.7	O3—C17—C18	125.2 (4)
C8—C9—H9A	119.7	O2—C17—C18	110.8 (4)
C9—C10—O1	126.1 (3)	C17—C18—H18A	109.5
C9—C10—C1	120.7 (4)	C17—C18—H18B	109.5
O1—C10—C1	113.2 (3)	H18A—C18—H18B	109.5
O1—C11—C12	107.3 (3)	C17—C18—H18C	109.5
O1—C11—H11A	110.3	H18A—C18—H18C	109.5
C12—C11—H11A	110.3	H18B—C18—H18C	109.5
O1—C11—H11B	110.3	H1WB—O1W—H1WA	107.3
			
C10—C1—C2—C3	−179.9 (3)	C11—O1—C10—C1	−172.2 (3)
C6—C1—C2—C3	−0.6 (5)	C2—C1—C10—C9	179.6 (3)
C1—C2—C3—C4	−0.1 (6)	C6—C1—C10—C9	0.2 (5)
C2—C3—C4—C5	0.7 (7)	C2—C1—C10—O1	0.4 (5)
C3—C4—C5—C6	−0.4 (7)	C6—C1—C10—O1	−178.9 (3)
C4—C5—C6—C7	179.6 (4)	C10—O1—C11—C12	−175.7 (3)
C4—C5—C6—C1	−0.4 (6)	C17—O2—C12—C11	−78.9 (4)
C10—C1—C6—C7	0.2 (5)	C17—O2—C12—C13	159.2 (3)
C2—C1—C6—C7	−179.1 (3)	O1—C11—C12—O2	−64.4 (4)
C10—C1—C6—C5	−179.8 (3)	O1—C11—C12—C13	54.0 (4)
C2—C1—C6—C5	0.9 (5)	C14—N—C13—C12	170.7 (3)
C5—C6—C7—C8	179.5 (4)	O2—C12—C13—N	−72.0 (3)
C1—C6—C7—C8	−0.5 (6)	C11—C12—C13—N	165.1 (3)
C6—C7—C8—C9	0.3 (6)	C13—N—C14—C15	161.4 (4)
C7—C8—C9—C10	0.1 (6)	C13—N—C14—C16	−73.6 (4)
C8—C9—C10—O1	178.6 (3)	C12—O2—C17—O3	4.0 (5)
C8—C9—C10—C1	−0.4 (5)	C12—O2—C17—C18	−176.2 (3)
C11—O1—C10—C9	8.8 (5)		

### Hydrogen-bond geometry (Å, º)

**Table:**

*D*—H···*A*	*D*—H	H···*A*	*D*···*A*	*D*—H···*A*
N—H0*A*···Cl	0.90	2.37	3.194 (3)	152
N—H0*A*···O2	0.90	2.59	2.948 (3)	105
C15—H15*B*···O1*W*^i^	0.96	2.53	3.469 (9)	166

Symmetry code: (i) −*x*+1, −*y*+1, −*z*+1.
